# The Constipation Habit

**Published:** 1884-07-20

**Authors:** W. J. Fairfield


### THE CONSTIPATION HABIT.

By Dr. W. J. Fairfield.

**A paper read before "The Calhoun County Medical Association," December 4th, 1883.**

The subject of constipation is so extensive, involving the discussion of so many diseases and remedies, and with its diarrhœa of literature covering so much ground, that I forbear, for want of time, if nothing more, from entering into a full consideration of the subject.

That it is often a symptom of disease, or a disturbance arising from disease, I need not discuss; but I wish, at this time, to call brief attention to it as a disease in and of itself, in order to elicit discussion, and thereby enlarge our ideas.

The constipation habit is certainly a perversion of an important function, and is often productive of great harm and suffering. The normal act of defecation, as a rule, occurs regularly once every twenty-four hours, and with a majority in the early part of the day, before or soon after breakfast. In health the call to evacuate the bowels is a peculiar sensation that cannot be misunderstood. If not heeded it may soon cease, and the call not return for an indefinite length of time. Immediately preceding this sensation is the peristaltic contraction of the sigmoid flexure which ejects its contents into the rectum, from which arises the warning and call for voluntary muscular assistance, that is so often unheeded or put off to a more convenient season. But the rectum must be relieved, and if not in the natural way then anti-peristaltic action takes place and the load is sent back whence it came, a burden and clog, blunting that delicate sense of the bowels.

Women, I think, neglect this function more than men. This is often from a false sense of modesty, their natural delicacy leading them to endure while away from home traveling or in society, rather than to withdraw, with eyes upon them, to a strange shrine devoted to *cloacina*. Even at their own homes, where there is a lack of modern conveniences, the inclemency of the weather, the exposure to cold and the foul breath of the privy vault, cause so much dread of the simple act of defecation as to lead them to procrastinate, to the utter demoralization of the normal defecative act. I have no doubt that the trammels of fashionable clothing also interfere to some extent. The considerable straining which is sometimes required to complete the act may be unattainable, from the clothing limiting too much the action of the diaphragm and abdominal muscles.

Sedentary habits which deprive the bowels of the gentle stimulus of exercise is one cause of constipation; and when to sedentary habits is added position or posture which cramps and crowds the bowels, as is the case with the shoemaker, habitual constipation is almost sure to follow.

The abuse of cathartics is a fruitful cause to induce and confirm this habit. What with the anti-constipation pills, wafers and pellets flooding the land to dredge the *primæ viæ* on the first indication of its filling up, or to be used from the fear that it will fill up, it is a wonder that nature's *cloaca* is maintained at all.

Errors of diet, though not mentioned first, are not least in causing this habit, which is, perhaps, more prevalent in this country than in any other; and some one has said that it is because we eat too little soup. Water, as a solvent and a diluent, acts in the alimentary canal a very important part, and soup-eating should certainly be encouraged in order to counteract the tendency to take our food too solid, and to favor the fæcal current.

Whatever line of diet we are in the habit of taking, and the bowels are normal, if we make a sudden or marked change in our diet, it is often attended by bowel disturbances in one way or the other. I have been in a position to observe a great many persons who have made sudden changes, particularly from a mixed, generous diet to a vegetarian diet, which, from its bulky nature, imposes more work on the bowels than they are used to, often beyond their working capacity, and the result would often be acute constipation. The next step, then, was to use the much-abused water enema, which to the overworked bowels seemed a God-send, but, by frequent repetition, proved a blight to their work, making them a sluggard in the human economy.

I give one case to illustrate:

Mr. S. had been a vegetarian for five years or more, and had adopted two meals a day. He was in fair general health for one of such habits, but his great difficulty was no natural action of the bowels, which had existed for the last five years. His sole reliance for a movement was the coarse food and water enemata, which he had come to take regularly.

He consulted me ostensibly for hemorrhoids, which he said the doctor who had treated him told him he had had, and who expected to operate on him. On making a thorough exploration of the rectum, I was not surprised to find no hemorrhoids, for he gave no symptoms of any. I found, however, a very large, pouch-shaped rectum, with flabby, relaxed and attenuated walls, which I attributed to the protracted use of the water enemata.

I changed his diet, stopped the enemas, gave him three meals a day, had him drink four or five goblets of water per day, and had him inject, on retiring, one-third of a cup of cold water to be retained. Ordered daily massage and kneading of the bowels, with a mild faradisation of the same; also, ten drops of fld. ext. casc. sag. four times a day. In four weeks time he had natural stools, without the use of medicine or treatment of any kind.

A too concentrated diet may cause this habit, but I have observed no danger in this direction. A variable appetite which makes extremes in quantity and quality of food is sometimes a cause, but, as this would lead us to discussion not intended at this time, we desist. I have often observed that a long journey by rail will produce a severe constipation, and have wondered if the constant jarring of the cars has anything to do with it.

The more difficult a disease is to treat successfully, the longer the list of remedies employed; and, judging from the length of the list in this case, one would be almost discouraged from attempting a cure.

Yet, with clear ideas of causes, the indications for treatment are simple; and, with the hearty co-operation of the patient, the physician may feel quite certain of gaining, sooner or later, the desired result.

The following I give as a general outline of the treatment, which, of course, must be varied somewhat according to the special indications of each case:

Regulate the diet, having three meals per day of palatable, nutritious food, not too bulky or too concentrated. Have soup at one meal each day.

On rising, at least an hour before breakfast, drink one or two large goblets of water. If stomach is weak and inclined to chronic gastritis, I order the water to be drank hot. Twenty or thirty minutes following the water, give the bowels a thorough kneading for ten minutes: then, assuming erect position, with arms above the head and left foot on a line with the right and placed in front of it, bend forward till the knuckles of the closed hands touch the floor, then back to the first position, repeating this five or six times; then, reversing the position of the feet, repeat the movements. This is an excellent exercise for the abdominal muscles and an inactive liver.

At night, also, before retiring, drink a goblet of water, and if there are indications of dryness of lower bowels, I use an enema of one-third to one-half cup of water, to be retained.

Flushing the sewer may be a good practice with some, making the stomach the flooding tank; but we must use great care not to interfere with digestion.

When it is available, I often order a fifteen minutes' daily application of electricity to the abdomen, using the Faradic current.

If any medicine is demanded, the first on the list is cascara sagrada. I think it is an excellent "peristaltic persuader." It renders, in my hands, the most efficient service in small and repeated doses.

I impress it upon my patients to make it a daily practice to go to stool at a regular hour, to induce, if possible, by voluntary muscular effort, a movement, remembering that this measure alone, if persisted in, will oftentimes overcome this deplorable habit. Perhaps the best time of the day for this is soon after breakfast. Patient continuation in this line of treatment will do a great deal to dispel this *bete noir* of medical practice.—*Detroit Lancet.*

